# Reading Jeffrey C. Alexander, Part I: The case for canonization

**DOI:** 10.1177/07255136261464660

**Published:** 2026-07-13

**Authors:** Celso M. Villegas, Galen Watts

**Affiliations:** 1 3475 Kenyon College, USA; 2 8430 University of Waterloo, Canada

**Keywords:** Jeffrey C. Alexander, cultural sociolgoy, neofunctionalism, classical sociology, canon

## Abstract

For nearly half a century Jeffrey C. Alexander has devoted himself to the sociological vocation − first as a general (‘grand’) theorist, and then as the iconic leader of a set of global research programmes − a life lived for sociology. In ‘Part 1’ of this two-part article, we make the case for Alexander's canonization as a classical theorist. We do so by reconstructing and periodizing his life and intellectual development, narrating his transformation from Jeff to ‘Jeffrey C. Alexander.’ We do this at the level of what Parsons's termed *personality*: that is, we situate Alexander's oeuvre in the quality of his person, his wants and needs, his frustrations and anxieties, and the development of the self.

## Introduction

Jeffrey C. Alexander has proven himself worthy of the highest recognition in sociology: the status of a classical theorist. Alexander's work on Parsons reveals two paths towards becoming a canonical theorist. One is *structural*: locating Alexander at the core of a research programme (see for instance Alexander and Colomy et al., 1998). The second is *cultural*: turning ‘Jeffrey C. Alexander’ into a condensed symbol, a shorthand for a complex set of ideas (Alexander, 1987a; 1998: 4fn6). No doubt any case for canonization will include both these dimensions and ours is no different. But to these we add a third tack. In this − the first of a two-part article on the life and work of Alexander − we reconstruct Jeff* the person* amid becoming ‘Jeffrey C. Alexander.’ We do this at the level of *personality*: we situate his oeuvre in the quality of his person, his wants and needs, his frustrations and anxieties, and the development of the self (Levinson, 1978). ‘Theorists present themselves… as guided by purely rational cognitive and rational ways,’ Jeff writes, reflecting on Durkheim's anxieties of being misunderstood (Alexander, 1986b: 198). We *know*, as sure as Jeff certainly does, that his ideas and work have an autonomy and salience of their own. We *feel* that such ideas and the encounters that formed them contain ‘emotionally laden and highly personal meaning[s]’ (Alexander, 1986C: 198). There is tension and frustration in misunderstanding, and those feelings are ‘provocative of further theoretical change’ (Alexander, 1986c: 198) – feelings that Jeff himself has channelled into molten works of theory. At the same time, it is possible that theory – really all work of the mind – can only truly bloom when our emotional needs are met, and our minds can be set free. This internal world of feelings – both negative and positive – may only appear as external response to others and remain opaque to the theorist themselves. And certainly, the structural and cultural levels of canonization will matter in our story. But in seeing how some of Alexander's work emerged from this ‘internal environment of action’ (Alexander, 1987b), we can better understand the value of his intellectual action and derive lessons for why we canonize a theorist beyond their structural roles and symbolic value. So, in what follows we periodize his life and intellectual development, narrating his transformation from Jeff to ‘Jeffrey C. Alexander.’

## From Jeff to ‘Jeffrey C. Alexander,’ 1947–1978^1^

Jeffrey Charles Alexander was born on 30 May 1947 in Milwaukee, Wisconsin. Jeff's parents could trace their lineage to culturally and socially distinct communities in the Jewish diaspora. His father, Frederick Charles Alexander, an ad man of German−Jewish descent who, though brilliant and comfortably middle-class, took little interest in high culture or the rarefied intellectual world his son would eventually summit. His mother, Esther Leah Schlossman Alexander, of Polish−Jewish and Russian−Jewish background, earned a Master's in Social Work from the University of Wisconsin, yet typical of the times, stayed home to care for both Jeffrey and his younger brother, Charles ‘Skip’ Alexander. Jeff and Skip went to a Reform temple Sunday School each week, but the Alexanders were a secular Jewish family. Fred and Esther considered themselves enlightened and assimilated in comparison to their parents. They had a house in the Fox Point suburb of Milwaukee, one of many Ozzie-and-Harriet-type middle-class households there. Agnostics, Fred and Esther understood their distance from Judaism as a measure of their family's assimilation into the American mainstream.

Indeed, Jeff's childhood was an all-American one. In addition to competing with Skip as brothers often do, Jeff played football, basketball and baseball at Fox Point School in 7th and 8th grades, then tennis and football at Nicolet High School. When Jeff was 15, the Alexanders moved from Milwaukee to Los Angeles. The change was consequential for the family's sense of assimilation. The Alexanders stopped sending their children to temple. Skip grew out his hair. And becoming a California Boy, Jeff traded American football practice in the upper Midwest for the ‘hottest, smoggiest weeks of the summer’ ((University High School) Education Foundation, 2012: 17) at University High School in West Los Angeles (LA). Jeff's two-way play at right guard and middle linebacker earned him league honourable mention all-league his sophomore year as a member of the school's B-Team. Though he disappointed his coach by quitting football, Jeff continued to play varsity tennis all three years at university.

The Alexanders arrived in Southern California amid the political change and optimism of the early 1960s. Fred and Esther had always been loyal Democrats; they were terrified of McCarthyism and enthusiastic supporters of Adlai Stevenson. In (LA), Esther hosted political events for Democratic candidates in the Alexander home, and she volunteered in the Watts neighborhood of Los Angeles as part of Great Society initiatives, often bringing Jeff along. Wanting a new camera, Jeff's father told him he should get a job, and so he did: at the Los Angeles Country Club. Back then, club membership was restricted − the Alexanders could never have been members because of their Jewishness − but ‘Alexander’ did not raise any alarms, not certainly for a teenager working back-of-house. There Jeff worked alongside Black cooks and waiters − the only white kid in the kitchen, and maybe the only Jewish person on the premises.

In a retrospective essay, Alexander describes his high school self as a model of ‘this-worldly ascetism and disciplined self-control’ (Alexander, 2018a: 101). At the same time, Jeff grew to love literature and writing under the tutelage of longtime University High English teacher Fred Holtby. Jeff remembers Holtby as ‘a brilliant and bohemian English teacher who made me and many other conventional West L.A. boys and girls into more thoughtful and more aesthetically, wide-awake individuals’ ((University High School) Education Foundation, 2011: 12). Holtby decorated his classroom walls with giant photographs of the ‘New Critics.’ Holtby's classes were intense, but liberating − free association writing, metaphorical essays in comparative literature − enough to inspire Jeff to want to become a writer. Transformed from within, Jeff submitted a piece to *The New Yorker* about a Black waiter whom he had befriended at the LA Country Club. *The New Yorker* rejected the essay, but sent back an encouraging note to the young Jeff to keep writing.

This combination of a studious self-control and a writer's sensualist spirit got Jeff to Harvard in 1965. He enrolled in Harvard's Social Studies programme, an interdisciplinary major where he took classes in political philosophy, literature and social science. His roommate was Chris Wallace, the son of acclaimed American journalist Mike Wallace. Yet, by his own admission, Alexander found Harvard − both in coursework and social life − quite hard. Though about 100 of Jeff's class of 1200 or so were Californians, there were even fewer other Jews. He also felt academically unprepared; many of his fellow students were graduates of East-Coast preparatory schools, whose families were rich in what Pierre Bourdieu (1984) reductively calls ‘cultural capital.’ Alexander, by contrast, lacked the necessary cultural know-how or know-that. Calling up his mother Esther, she told him, ‘If you’re unhappy, you should talk to someone about it.’ He started talk therapy in the summer after his second year.

Therapy helped, but Alexander really only began finding his footing at Harvard as he began writing for the school's newspaper, *The Harvard Crimson*, which became a central focus of attention during his sophomore year. Returning home for the summer of 1967, Jeff got a job at the *Los Angeles Times* as a copy boy. When the drudgery started to get the better of him, he marched into his boss’s office and made an ask: the *Times* had a beat for classical music and for pop music, but not for rock. Could he go to a few gigs and write some pieces? As it turned out, his boss had also lived in Jeff's residential house at Harvard when he was a student (now cultural and social capital were working in Jeff's favour) and he decided to give Jeff a chance.

That summer the 21-year old Jeff, with a *Los Angeles Times* press credential, would attend shows at Whiskey-a-Go-Go in Hollywood on Friday nights and then would interview the acts the very next morning − neither reporter nor band members getting much sleep. One of the first of these interviews was with the psychedelic rock band The Doors, led by their charismatic frontman, Jim Morrison. See if you can detect the difference between the eager seriousness of the cub reporter and the… altered states of his subjects:

(Interviewer: Dope is a common point of reference in your songs.)

Jim [Morrison] – ‘Oh no, really? Wait a minute what's dope? Name one song that mentions dope.’

Ray [Manzarek, organist] – ‘Not really, there's no reference to drugs.’

John [Densmore, drums] – ‘Well, there are a whole lotta levels you can get it on.’

[…]

(Interviewer: Are drugs an important part of the creative process?)

Jim – ‘About drugs or anything else, I think everybody should do what they want, that's all.’

Ray – ‘Whatever the individual has to get those inner feelings out is great. Drink, smoke, meditate, any one of a million things’

Jim – ‘Even celibacy.’ (Alexander, 1967a: D53)^2^

Reporting was not all fun and games. 1967 would see an escalation of anti-war protests, and Jeff reported on protests in the LA area following the Glassboro summit between US President Lyndon Johnson and Soviet Premier Alexei Kosygin (Alexander, 1967c). Upon returning to Harvard for his junior year, Jeff wrote a frenzy of stories, covering campus goings-on; human interest pieces about Harvard kids trying to become rock stars; and writing long form investigations such as a two-parter on the moral condition of Wellesley girls, and a three-part piece on the Evelyn Wood reading school (which was fleecing Harvard students by purporting to teach them how to speed read). Alongside those pieces, the *Los Angeles Times* would publish Jeff's interview with Buffalo Springfield and the *Crimson* his interview with Jefferson Airplane. In other words, Jeff had found his first vocation. Truly prolific, every word seemed to matter to the young Jeff in the way that every word might matter for someone feeling like they could write themselves into adulthood.

Consequently, Jeff finding his voice in journalism in 1967–1968 would dovetail with what Parsons and Platt (1973) would later describe the ‘expressive revolution’ in American society and the increasing tumult of the student movements of 1968 around the world. Both the Alexander brothers became spellbound by the radicalism pulsing through campus and the wider society, in their eyes the New Deal liberalism of their parents was now consigned to the dustbin of history. Jeff's writing for the *Crimson* took a decidedly political turn. In early 1968, he filed a story in the *Crimson* about Harvard students helping working-class Boston kids dodge the draft (Alexander, 1968). His brother Skip, who joined Jeff at Harvard in 1968, was enthralled by the Mararishi Mahesh Yogi and with transcendental meditation − which Skip continued to practise throughout his life (see Alexander, 2000a).

The Spirit of 1968 would forever mark Jeff's intellectual and political understanding. In the autumn of 1968, his senior year, Jeff joined Harvard's branch of Students for a Democratic Society (SDS). Like other ‘sixties communard[s]’ (Alexander, 2018a: 102) possessed by the ‘spirit of socialism’ (Alexander, 2010), the young Alexander craved a socialist revolution. He and his comrades agitated inside and outside of the classroom, railing against the hypocritical liberalism that led the United States to war in Indochina as well as the conservative nature of Harvard's student life policies. Jeff traded in being the author of so many stories in the *Crimson* for being their subject, calling for democratic reforms of standing committee membership, Reserve Officers’ Training Corps recruiting, and to Harvard's parietals policy (which prevented men and women from being in the dorms together after 10pm), as much as thoroughgoing socialist revolution.

The Marxism that attracted Alexander and his New Left comrades was one that called for direct action, but also was opposed to the economistic and scientistic Marxism institutionalized in the actually-existing socialist societies of the twentieth-century. In other words, he and his comrades venerated the early humanist Marx (Gouldner, 1980) whose writings on alienation and *species-being* reverberated the counter-culture's humanistic concerns with subjectivity, consciousness and democratic participation. Alongside H. Stuart Hughes and Michael Walzer's assigned readings from the classics of liberalism for his Social Studies degree, Jeff and his comrades − among them the historian Michael Kazin − read on their own Sartre, Marcuse, Paul Goodman, Andre Gorz and E.P. Thompson. The revolution would be one of feeling as much as material transformation, it would be one of solidarity and independence of mind and spirit. It would come from the ground up − from the ‘new working class’ and its allies − not from The State down, and it would be democratic in its practices.

These intense experiences of activism, and Marxist theory led Jeff to a new dream of adulthood: graduate school to, in his words, ‘become a socialist intellectual.’^3^ Inspired by C. Wright Mills, the rebel sociologist who coined the term ‘New Left’ and author of *The Sociological Imagination* (Mills, 1959), Jeff had his first encounter with the man whose work would propel his early career: Talcott Parsons. After applying to graduate school in sociology in the autumn of 1968, Jeff realized it might be a good idea to take at least one course in sociology before leaving to get his PhD. He chose a theory course taught by Parsons. On the day Parsons was scheduled to discuss power, Jeff stood outside the door of the classroom distributing a pamphlet he had prepared attacking the functionalist approach. As Parsons entered the classroom, he asked Jeff for a copy, read it in silence, and calmly addressed the assembled students: ‘Mr Alexander has expressed reservations about my theory of power. Rather than taking up classroom time to respond, I invite anyone who would like to discuss these points in detail to my home next Tuesday evening.’ Parsons having defused the tension in the room left Jeff a bit embarrassed and a little furious.

In the crucible of 1968, Jeff fused his activism, ideology and hisincreasingly confident and prolific writerly talent into an honours thesis. Titled, ‘Violence and the American Labor Movement: 1865 to 1895’ (Alexander, 1969), it was a meditation on the meaning of political violence on the conservative tendency of American labour activism. His advisor was Barrington Moore Jr, an old money scion, Russian specialist and friend of Marcuse, whose classic *Social Origins of Dictatorship and Democracy* (Moore, 1966) would inspire a left-Weberian and comparative-historical trend in sociology − a trend Jeff would eventually see himself opposing. As Jeff was writing his thesis, Moore would publish a commentary ‘Thoughts on Violence and Democracy’ (Moore, 1968) which highlighted the contradiction between the purported values of American democracy and freedom with the violence perpetrated against Black rioters at home and on Southeast Asia: ‘…a violence that the powerful exercise against the weak in the name of freedom and order, a time-honored practice in Western culture,’ Moore writes (Moore, 1966: 2). The ‘Dark Side of Modernity’ (Alexander 2013) was plain to see − for both Moore and Alexander. Violence in the name of freedom could not sustain a democratic life, and for the young Jeff this contradiction indicated the end times of capitalist democracy. Brutality revealed the nakedness and corruption of the world, only revolution from below could bring about a world without contradiction.

Some dogged determination was necessary to keep this dream alive in the summer of 1969. Rejected from the University of Chicago, and accepted at Berkeley but with no funding, Jeff spent the summer supporting the Black Panthers at the Roxbury Collective in Boston. In June of 1969, he and his comrades travelled to Chicago for the SDS National Convention. The convention was tumultuous. The Workers−Student Alliance brandished their Little Red Books, while Jeff's Roxbury comrades joined the Revolutionary Youth Movement faction − soon to become the Weathermen. Upon returning to Boston, many of the Collective who like him had been accepted to graduate school declined their offers to continue the revolution via armed struggle if necessary − except Jeff, leaving him ostracized. Jeff left Boston disillusioned with both the vanguardist and violent turns in the SDS.

Berkeley was like Harvard in that Jeff found intellectual comradeship, but his radicalism would continue to suffer blows. Violence and pessimism occluded the path to socialist revolution and the end of the Vietnam War. The violent removal of protesters from People's Park in May of 1969 preceded Jeff's arrival. In his first year at Berkeley, Jeff became a junior participant in James Weinstein's *Socialist Revolution* (later *Socialist Review*) editorial group (which included John Judas and Eli Zaretski) and followed the lectures in Marxist philosophy of Richard Lichtman. Jeff also became fast friends with fellow graduate student Fred Block. In spring 1970, Nixon ordered an invasion of Cambodia, sparking protests around the country, the black mark of that year being the shooting deaths of four students by the National Guard at Kent State University. Jeff's collective travelled to LA to help with a unionization drive at a Goodyear tire plant, offering to write propaganda. They made up a little ideological poster for the union, and then were told their help was not really needed. Jeff and Fred then organized Berkeley faculty, students and graduate students to canvas for working-class support for the antiwar movement in Fremont, California, then the home of a General Motors factory. After three months of going door-to-door, Jeff was ‘dumbfounded by the fact that these guys [had] small swimming pools in their backyards and lived in nice little houses’ and saw no problem with their lives as they were − not exactly the alienated working class his theory had led him to believe. At the same time, the revolutionary vanguardist streak that pushed Jeff away from SDS had not abated, and among would-be leftist allies, violence seemed to be the only way out.

The 1970s challenged Jeff's socialist self-image both intellectually and politically. 1971 saw the mass publication of Gramsci's *Prison Notebooks* in English. Jeff tried to link hegemony to collective consciousness in a paper for Leo Lowenthal's course on Durkheim, to which Lowenthal apparently said, ‘Why would you do this? What kind of crazy paper is this, Jeff?’ Doubts began to creep back into Jeff's psyche: could he continue to define himself as a radical intellectual? He began psychoanalysis − a practice he continues to this day − his second year of graduate school, hoping to map the deep trenches of his hatred of capitalism but his abhorrence of violence. In 1972, the Watergate scandal made national headlines and instead of hearing a Gramscian battle of hegemony between the representatives of the bourgeois classes, Jeff first noticed common, almost universalistic themes in their speech. Republicans and Democrats both made claims about the obligations of office and the higher purpose for their task, namely to preserve democratic institutions. Nixon was out and Jeff was elated, but this raised new questions. The Jeff of 1968 who ‘voted with his feet’ against formal democracy and the Jeff of 1972 who canvassed for McGovern stood in stark contrast to each other. If structure could not by itself explain Watergate, could culture as a relatively autonomous thing? And if so, why would a socialist such as him feel so inspired by the higher ideals of bourgeois democracy?

Jeff needed new intellectual resources to explore these new structures of feeling, new emotional supports that could permit him to make a break with how he had defined himself thus far. It would mean taking seriously the bourgeois science of sociology, something he was luckily already there at Berkeley to do. In addition to Lowenthal's honesty which he would rely on from then out, Jeff found lifelong mentors in Neil Smelser and Robert Bellah. While all three men would later sit on Alexander's doctoral committee, it was Smelser and Bellah (both students of Parsons), who had the most formative impacts on the young Alexander. In Smelser, Jeff would find a kindred *wunderkind*, and an exemplary model of how to integrate opposing ideas and theoretical perspectives in the service of producing a larger synthetic whole.^4^ Smelser was in many senses a traditional sociologist, an eventual president of the American Sociological Association and devoted to sociology as a discipline.

And in Bellah, Alexander would find a paradigmatic public sociologist, whose moral and intellectual passion would inspire Jeff's own, and whose ‘symbolic realism’ would later form the foundation of the Strong Program.^5^ Bellah was the opposite of Smelser in many ways: prone to denouncing this and denouncing that, whose mind and work travelled far afield from sociology. It was Bellah who encouraged Alexander to read the work of Talcott Parsons, starting in the summer of his second year at Berkeley, and then more intensely in a seminar with Bellah in the autumn of his third year, an experience he found revelatory. What most struck Alexander about Parsons's work was both the scope and ambition of his theoretical project, as well as its underlying normative commitments − commitments which, not coincidentally, aligned much more with his parents’ New Deal liberalism than with cultural Marxism.

*The Structure of Social Action* would unlock Jeff's mind so much more than the altered states of the 1960s ever did. Telling Bellah his seminar paper would be a bit late, Jeff wrote 105 pages on Marx and Marxism using Parsons’ approach to voluntarism and instrumental rationality. The paper, Jeff recalls, was perhaps deeply insane in its ambition but also the beginning of a true intellectual clarity: a moment of energetic release, a bridge from which he walked from Marx to sociology, distilled as best as it could into a paper for a mentor whom he knew would listen. Having poured himself into it, Jeff was a little annoyed that Bellah did not say anything about his paper for days. But when Bellah called Jeff on the phone, there was, relief, validation, and in more ways than one, permission to move on to the next part of his life. This is how Jeff remembers the phone call:

‘Jeff, this is Bob Bellah. I just wanted to let you know your paper is brilliant, you really have to know this is incredibly significant.’

‘Well, okay,’ Jeff recalls replying. ‘Do you think I could do my dissertation on it?’

‘Oh absolutely,’ Bellah said to him. ‘I mean, just expand it by another hundred or two-hundred pages.’

## ‘Parsons Jr:’ The anxiety of being misunderstood, 1978–1987

‘Theoretical Logic in Sociology’ the dissertation filed in 1978 is the (relatively) diminutive forebear to the four-volume *Theoretical Logic in Sociology* (Alexander 1982a, 1982b, 1982c, 1982d), whose final volume was published by the University of California Press in 1983. ‘Theoretical Logic’ is a hermeneutic deep reading of Marx, Durkheim, Weber and Parsons, assessing each on the degree to which they effectively answer the Problem of Order (whether social life emerges from interaction at the individual level or is created from already-existing collective structures) and the Problem of Action (whether social action is motivated by instrumental rationality or by nonrational considerations). All theory, Jeff argued, was founded on these basic presuppositions. Think of Order and Action forming a two-by-two table, with each dimension crossing each other like an x−y axis. While some great theory could get by on focusing on any one corner of the Order−Action matrix, any grand theory worth its salt would be multidimensional − successfully integrating the range of possibilities of order and action.

The narrative of ‘Theoretical Logic’ is surprisingly straightforward. Marx, for all his successes in elaborating a theory of capitalist exploitation, settled in a reductive way on a collective and rational basis for order and action, respectively. Durkheim, on the other hand, seeking to correct and at the same time run away from Marx, settles on a collective and nonrational view. Both Marx and Durkheim are reductionist in those regards, unable to effectively address individual voluntarism without hedging and stuttering. Weber, by contrast, has identifiable components of his thought that address all four quadrants in our two-by-two, but he never systematically worked out a way to synthesize the elements together. Parsons indeed succeeds at elaborating a theoretical system that does take all possibilities of action and order into account, but as he elaborated his system, he grew more and more abstract, and as he did, his work was thoroughly misunderstood by critics who could not grasp what multidimensionality was in the first place. The upshot is that whatever theoretical movement will come after Parsons, it should not discard Parsons’ ambition for a multidimensional sociology, providing nonreductive answers to both the Problem of Order and the Problem of Action.

Given the freedom to theorize at a grand scale, Jeff could now grasp the field as a whole for the first time. ‘If sociology could speak,’ he wrote in Volume 1 of *Theoretical Logic*, ‘it would say it was tired’ (Alexander, 1982a: 1). Indeed, by the time ‘Theoretical Logic’ was filed, sociology was suffering from a malaise. Parsons was out − the structural−functionalist project Parsons had built fell like a Tower of Babel, with critiques of his work evolving into new branches of the field. The left-Weberian turn in theorizing and sociological analysis came of age in the late 1970s, with figures such as Charles Tilly, Michael Mann and Theda Skocpol rising to prominence − all three taking a structural and historical approach, eschewing individual actors for the power of the state and the balance of class forces. The discipline's love affair with statistical analysis did not abate but neither did its empirical qualitative traditions. It turned those positivist powers towards social problems, taking on board C. Wright Mills’ admonitions from the late 1950s: abstract empiricism was out, more prosaic issues of race, class and gender discovered in surveys, content analysis and ethnographic work were in. If C. Wright Mills first took aim at sociology's sickness in *The Sociological Imagination* (Mills, 1959 [2000]), it was Alvin Gouldner whose *The Coming Crisis of Western Sociology* (Gouldner, 1973) foretold of Jeff's cohort of young sociologists coming to save the discipline from its scientism and its inability to effectively pose questions of itself. Reflexivity became a central plank of theorizing, and despite his own critiques of Parsons, Gouldner found Jeff's multidimensional approach to be right in line with the self-critique the field needed. After meeting Jeff at California State University at Northridge – where Jeff was a critical respondent to Gouldner's newest book – Gouldner invited Jeff to the editorial board of his new journal, *Theory and Society. *

By the end of Jeff's seventh year at Berkeley in 1976, things were looking up. Jeff was teaching his own courses in theory, a privilege for a graduate student, and he was locking up a job at the University of California, Los Angeles (UCLA), where he would work from 1976 to 2001. In addition, his dissertation was generating significant buzz. The University of California Press got a hold of ‘Theoretical Logic’ and wanted Jeff to turn it into a book. His editor, Grant Barnes, said the outside reviews were great and all the dissertation needed was a new introduction. Easier said than done. The entire text needed to be reframed. The introduction needed to set the argument up against the history of the philosophy of science to give readers a grasp of the importance of multidimensionality and its presuppositions. As the 1970s wore on to the early 1980s, Jeff kept editing and writing but frustration and embarrassment set in as the word count grew. Barnes was generous and encouraging. Jeff apologized for the now roughly 3000-page book project − it would need to be split up until four volumes, each released as he finished the edits to each section. Gouldner also encouraged Jeff in his phone calls, telling him the book was important and he should get it done. As Gouldner, Barnes, Bellah, Smelser and others talked about Jeff's project, word-of-mouth spread at conferences that someone was writing a grand rereading of the classics, plus Parsons.^6^ There was anticipation for *Theoretical Logi*c: it was supposed to make a splash.

But with anticipation, there came an intense scrutiny when *Theoretical Logic*'s individual volumes were released. The reviews − of which there were many − ranged from reverential to scathing and everywhere in between. The older generation of theorists appreciated *Theoretical Logic* far more than those of Jeff's generation. Perhaps it was from the audacity of the task itself − a move to grand theory when most of, if not all, of American sociology had left it behind − and that Jeff had the gumption to do it. Perhaps it was the provocative language of *Theoretical Logic*, the thoroughness of its critique of critiques that impugned leading lights in the field for failing to see what Jeff could with his metatheoretical framework. Perhaps it was just the serious attention paid to Parsons. Only someone who ignored the trends in the discipline could do this, given Parsons’ now-polluted status: ‘Parsons, Jr.,’ as Alan Sica put it (Sica 1983). Or perhaps it was the audacity of the physical form of each volume: a dapper photo of the young Jeff on every dusk jacket, a ribbon bound to each volume’s spine as if it were a Bible, almost declaring it a classic (and its author a hotshot) before the rest of the discipline could have a say.^14^ "[Theoretical Logic] has thus become not so much a book as a symbolic event," in the words of Robert Alun Jones (Jones 1987). Meeting expectations for scholarly output was never an issue for Jeff the prolific writer: he was tenured and promoted to Professor at UCLA in 1982. Meeting expectations to continue the miraculous works of theory in the vein of *Theoretical Logic* and its bold author ‘Jeffrey C. Alexander:’ this was the true test.

To that end, Jeff refined one of his reputation-defining skills: agenda-setting via a polemic reframing of arguments and authors. Presuppositions and multidimensionality offered a means of passing judgement in a consistent way, for ‘*every* theory commits itself,’ ‘assumes,’ or otherwise ‘contains’ some form of address pointing to the order and action questions (Alexander, 1979a: 345, emphasis added; Alexander, 1984b: 6–7). ‘The presuppositions of *any* social theory are the positions which a theory takes about the nature of human action and the manner in which plural actions are interrelated’ (Alexander, 1985b: 26, emphasis added). On the one hand, such claims do logically follow from a metatheoretical toolkit; on the other hand, that *every* theory can be grasped as such sets the scale of Jeffrey C. Alexander's unique insistence that theory must be, should be read in this way.

Then, there is the taking the tale-of the-tape of other players on the field of theory, again practised first in *Theoretical Logic* and now marking the Alexander ‘brand.’ In a review of Parsons’ career, published in *The New Republic*, Jeff (Alexander, 1979b) writes, ‘Parsons did not enjoy a good fight.’ This does not seem to be true of ‘Jeffrey C. Alexander.’ Jeff calls Jürgen Habermas a ‘brilliant, but flawed thinker’ who commits an ‘error on the theoretical and presuppositional level itself’ in which he ‘does not go quite far enough’ to distance himself from his Frankfurt School-cum-Marxian inheritance (Alexander, 1985b: 413, 423). For all the honesty of George Homans’ autobiography, Jeff judges Homans to have ‘used this unique genre to conceal as to reveal,’ full of ‘retrospective distortion’ of Homans’ true allegiance to the individualist perspective (Alexander, 1986a: 446, 447). On the one hand, no one would dispute that critique can be incisive; on the other hand, there is a consistent sort of argument that the theorist under review is opaque to themselves and to their own presuppositions that only ‘Jeffrey C. Alexander’ could reveal.

Even if Jeff could be distinguished from Parsons in his argumentativeness, it was the symbolic and intellectual link to Parsons in *Theoretical Logic* that was as much an opportunity as a constraint onto Jeff's early career. In terms of its culture−structural opportunities, if ‘Parsons’ was a polluted symbol in the eyes of most American sociologists, Jeff would therefore attempt an intellectual rite of purification. Reading Parsons with faith amid the suspicions of the day, Jeff would defend Parsons against ‘reductionist’ readings. Contrary to the claims of the conflict theorists, Parsons did have an elaborated model of social change that marked him not as a conservative but a social democrat (Alexander, 1978, 1981). Parsons’ intellectual descendants had actually succeeded in preserving the core of his work not via blind adherence but by revising his claims against their own biographical, historical and intellectual interests (Alexander, 1979a). Contrary to their American counterparts, European theorists − particularly in Germany − had explicitly built on Parsons rather than throwing him out (Alexander, 1984b). And to the book that opened his mind to Parsons back in 1973, Jeff celebrated *The Structure of Social Action* for setting the foundational agenda for nearly all the major theoretical developments of the twentieth century (Alexander, 1988b).

Teaching at UCLA facilitated a move from the high theoretical language of *Theoretical Logic* to a more accessible form: Jeff's second classic book, *Twenty Lectures: Social Theory Since World War II* (Alexander, 1987b).^7^ Like all good lecturers, Jeff tells us a story in *Twenty Lectures*, and in the palms of our hands we are transported to a UCLA lecture hall and then across the lives and times of theory and its theorists. He plots theory's development from *The Structure of Social Action*, to a post-Parsonian phase, characterized by the rise of antagonistic, yet uncoordinated, responses to Parsons's prior hegemony, such as left-Weberian macro-conflict sociology (Gouldner, Mills, Collins and Rex) more micro-level ethnomethodology (Garfinkel), symbolic interactionism (Blumer) and exchange theory (Homans). Finally, a third, ‘postclassical phase,’ characterized by the emergence of a new generation of sociologists − among them, Alexander and Collins in the United States, Giddens and Archer in the United Kingdom, Habermas and Luhmann in Germany, and Bourdieu and Boltanski in France − all interested in reunifying sociology by means of a grand theoretical synthesis that would transcend the classical antinomies of agency/structure, subjective/objective and micro/macro.

But now to the constraints. If Parsons had left behind a kingdom of grand theory, then Jeff was seen to have made a claim to ‘the throne that … stood unoccupied since Parsons's fall’ (Joas, 1988: 472). As one commentator put it years later, ‘If Parsons is the father of sociological theory, Alexander is one of his main heirs presumptive’ (Mouzelis, 1999: 721). Holding others to account on their presuppositions was one thing, and a defence of Parsons’ core contributions was fine in and of itself, but could Jeff create theory to match the ambition of Parsons’ multidimensional, field-defining work? Not just Parsons Jr, could Jeff achieve theory's crown as the Parsonian Prince? Indeed, the new generation of theorists Jeff acknowledged in *Twenty Lectures* and elsewhere included the leading lights of ‘contemporary theory’ − Giddens, Beck, Habermas, Collins, Bourdieu, among others − all contenders for the throne, all having published work in this period that staked a claim at uniting the warring tribes of theory into one.

Akin to neo-Marxism's relationship to Marx, ‘Neofunctionalism’ reconstructed Parsons’ work by carefully recovering its important contributions but opened itself up to other ideas and new empirical referents (Alexander and Colomy, 1985). The strong microinteractionist contingent at UCLA would push Jeff to consider more seriously the shape of the individual actor than even Parsons and action theory ever did. Jeff's first graduate student, Paul Colomy, already at UCLA studying symbolic interactionism, and the founder of ethnomethdology himself, Harold Garfinkel, were central in that concern. Social movements and the frontlash/backlash dynamics of Karl Polanyi's ‘double movement’ (Colomy, 1986: 149ff) explained pressure and conflict in a more concrete and vital way. What Jeff and Paul Colomy created was a ‘left’ version of Parsons’ differentiation theory that did away with evolutionary teleology, eschewed Parson's sociological idealism, could account for both equilibrium *and* conflict and which sought to build in collective actors into this model for change (see Alexander, 1985a; Alexander and Colomy, 1985, 1990). Alexander and Colomy declared neofunctionalism part of ‘The New Theoretical Movement’ in sociology, theorists willing to approach grand theory again in the spirit of synthesis (Alexander, 1987b; Alexander and Colomy, 1990).

The miracle announced, the masses awaited at the foot of the Mount. Like *Theoretical Logic*, ‘tongues have been wagging about the [neofunctionalist] movement's program which, according to gossip in the guild… [that] there will be a mighty demonstration, as Alexander has argued at length, that Talcott Parsons lives…[!]’ quipped Charles Page (1985: 10). But neofunctionalism was perhaps less miraculous. For one, neofunctionalism's more ecumenical tone contrasted strongly against Jeff's polemics for Parsons. Sure, there were ambitious declarations, but in practice Jeff insisted on highlighting others’ work − the *Neofunctionalism* edited volume in 1985 demonstrating this. ‘Less a theory than a broad intellectual tendency,’ Jeff wrote of the movement in the introduction to *Neofunctionalism* (Alexander, 1985a: 11): he could count on Parsons-era thinkers such as Smelser, S.N. Eisenstadt and Mark Gould; contemporaries of his, such as Richard Munch, David Sciulli, and Jeffrey Praeger; and Jeff's student Paul Colomy. But the incredulous had set the terms of the miracle: barring resurrecting Parsons himself, Jeff was expected to have transfigured himself into Parsons, not to be one of many.

Two, aiming not to demolish Parsons, but rather to carefully take apart his complex theoretical apparatus, assess each piece and build with its best parts, required an elaborate metatheoretical scaffolding − presuppositions, scientific continuum, postpositivist case for the classics, reconstruction, elaboration, revision, etc. Jeff claimed this scaffolding offered a sturdy worksite to stand outside of theory as such − not just Parsons − and to provide a standard for judging, extracting and rebuilding projects during the New Theoretical Movement. But was neofunctionalism the framework or the theoretical product? ‘I felt that neofunctionalism could be the platform for a satisfactory macro sociology,’ Jeff would say in retrospect (Cordero et al., 2008: 525). But he ‘felt the content bleed out of neofunctionalism in the early nineties and I ended my own involvement in the mid-nineties’ (Vandenberghe, 2019: 29): No doubt that scaffolding was a tremendous feat of the mind in and of itself, but without content neofunctionalism could only be just form.

Despite distancing himself from the project, Jeff would be strongly associated with it for the rest his career. ‘When I went to China in 2015, I realized the only work of mine that people knew was Neo-Functionalism,’ Jeff said in an interview in 2017 (Alexander, 2020: 75). In some ways, this was a good thing: clearly the case for neofunctionalism following functionalism was convincing and The New Theoretical Movement's reach was indeed global. Neofunctionalism's core texts were translated into German, French, Hungarian, Bulgarian, Brazilian, Italian, Spanish, Korean, Chinese, Japanese, Ukrainian and Russian. With Polish sociologist Piotr Sztompka, Jeff co-founded the International Sociological Association's Research Committee on Sociological Theory (RC16) in 1987, a move towards the institutionalization of the Movement at the transnational level. The ‘Jeffrey C. Alexander’ *the world* likely encountered was the Alexander of this era.

It was therefore United States audiences that were largely put off. In terms that would make sense to Americans of the day, Randall Collins described Jeff's plight from *Theoretical Logic* to neofunctionalism:

‘Alexander has been the victim of the Carl Lewis syndrome applied to sociology. Having had it announced in all quarters that one will win four gold medals in the Olympics, one becomes an emblem of arrogance that is hard to live down, whether you win four gold medals or not. In Alexander's favor one might say that he didn’t do the hyping himself, but had it done for him. But the result has been the same. Win or lose, he is put in the position where it seems nothing he does can win back the public favor now turned against him’ (Collins, 1985: 877).

Collins was insinuating something about the populist nature of American sociology at the time: less a question of the charisma of traditional founders, or anything about the validity of a postpositive case for their preservation, but more about how audiences fuse with their theorists like sports fans and their players (Taylor, 2022). Rumours and chatter at conferences like bar talk on game day, reviewers like sports commentators heightening the viewing audiences’ expectations, ‘Can Jeffrey recover from this?’ Collins asks a little facetiously. But he sympathizes here − he separates Jeff the actor and person from ‘Jeffrey C. Alexander’ the bundle of expectations (Collins, 1985: 877). The tragedy of neofunctionalism is the Agony of Defeat in this Wide World of American Theory. But Collins asks us to ‘Stay tuned’ − he knows there is more to come from Jeff, whose work he has now come to understand and respect.

Underneath and constrained by neofunctionalism were stirrings of truer, more vital interests that could not be put in Parsonian terms. In 1979, Jeff received a Guggenheim Fellowship to study the Watergate hearings at the Vanderbilt Television Archives. The lingering personal questions about his second political awakening in 1973 were now given some systematic empirical answers − ‘What I heard and saw was something like a code, detailed and yet at the same time generic, composed of layered sets of symmetrically equivalent binaries… I knew I had “discovered” something significant here… but I was not quite sure what’ (Alexander 2007: 645). ‘Three Models of Culture and Society Relations: Toward an Analysis of Watergate’ (Alexander, 1984a) owed much to Parsons’ analytical division between the cultural and social systems. But Watergate was not cultural ‘specification,’ but rather ‘refraction’ − the divergent class and party interests of Democrats and Republicans ‘[drew] on fundamentally similar cultural themes’ and evoked ‘the higher antitheses between the sacred and profane elements of American civic religion’ (Alexander, 1984a: 295, 309). Jeff was closer to the musicality of culture, but as if on cue, ‘Jeffrey C. Alexander’ appeared to conclude the piece ‘on a more general note’ about the ‘massive assault on functionalist theory [!]’ and the ‘path towards neofunctionalism’ (Alexander, 1984a: 311).

In 1984, Jeff attended a conference bringing together American and German theorists at the University of Giessen in West Germany, hosted by Bernhard Giesen who would become one of Jeff's closest friends and collaborators. Jeff and his family spent his sabbatical year 1985–1986 at the Institute for Advanced Study at Princeton, reuniting him with his old Harvard professor Michael Walzer. The paper Jeff crafted from all this, ‘Action and Its Environments’ (Alexander, 1987a), was part of the spirit of synthesis of The New Theoretical Movement, it drew on Parsons, and it was strongly influenced by Jeff's UCLA colleague Garfinkel. But it was remarkable in that it offered a solution to the action−order dilemma Jeff had posed in *Theoretical Logic*. Every action could contain both rational and nonrational elements − what Jeff now called strategization, typification and invention − because within and without the concrete actor were the ‘more or less ordered environments’ through which those actions took place: the internal environments of personality and culture; and the external environment of social structure. Still, Jeff was constrained by Parsons, provisionally including ‘values’in the catalog of contents of cultural system. ‘Action and Its Environments’ − not neofunctionalism − would be the real platform from which Jeff's own cultural sociology would be launched.

Perhaps the most consequential intellectual move involved finding a new theoretical patron saint to better understand the sacred and profane, culture and ultimately himself. ‘By the time I went to the Institute for Advanced Studies… I had become acutely dissatisfied with the work of Weber, the early Durkheim, and particularly Parsons’ theoretical multidimensionality.’ This meant going back to ‘the foundation, to the building blocks’ and ‘recovering semiotics, structuralism, post-structuralism, all in the spirit, if not the letter of the late Durkheim’ (Cordero et al., 2008: 525). Jeff returned to his interpretation of Durkheim in *Theoretical Logic*, that Durkheim had ‘really decided to start his sociology all over again… in the late 1890s, with his discovery of religion’ (Cordero et al., 2008: 525).

Reading Jeff's two-part 1986 return to Durkheim's intellectual development (Alexander, Alexander, 1986b, Alexander, 1986c), one could be forgiven for thinking the story was Jeff's too. Like Durkheim who had ‘trumpeted’ his first book *Division of Labour* as the beginning of a ‘new science,’ Jeff too had made his charismatic arrival onto the field with *Theoretical Logic* and trumpeted his neofunctionalism (Alexander, 1986b: 97). Durkheim's dissatisfaction stemmed from being unable to say what he valued − the freedom of the individual − amid the intellectual context of a ‘renewal of Marxism in French society’ which viewed his past work as speaking to the material constraints of social life (Alexander, 1986b: 100–102). Like Durkheim, Jeff's neofunctionalism was misconstrued in his eyes as a kind of conservative reaction to the sociology of the day rather than a way to engage with it. Durkheim was prone to vituperative defences of his work because he realized, ‘consciously or not, that the theory that informed so much of *Division* was a drastic mistake’ (Alexander, 1986b: 102–103) − Jeff was coming to this realization about Parsons, too.

Durkheim's path left clues for Jeff's future. An encounter with the work of Robertson Smith led Durkheim to the radical realization that ‘society could be determinate, organized, and voluntary at same time: collective order would be accepted because it held to be sacred’ (Alexander, 1986c: 191). Durkheim's ‘religious sociology’ had an ‘intellectual vehicle’ with his journal *L’Année sociologique* (Alexander, 1986c: 195). Durkheim's doubts, his wish to develop and ambitious, comprehensive sociology, the intellectual trends of the day and the pressure of expectations and criticism formed his ‘more immediate and personal intellectual environment’ − personality, culture and structure entered into his actions, but Durkheim was able to make an intellectual breakthrough (Alexander, 1986c: 198). Perhaps in a wish for himself, Jeff writes ‘Durkheim had finally differentiated his own theory from Marx's in a conclusive way’ (Alexander, 1986c: 195). For Jeff, this would mean coming to a deeper understanding of agency − both analytically in his work and concretely in his person.

## ‘Every action contains a dimension of free will:’ 1987–2001

In 1983, the album *Colour by Numbers* by Culture Club hit number 1 in the United Kingdom and number 2 in the United States. An R&B band among the British New Wave and styled in New Romantic aesthetics, Culture Club was an emblem of the MTV era when fresh sounds and striking visuals opened up the *bricoleur*'s imagination. The music video for their hit song ‘Karma Chameleon’ is set on a casino steamboat in Mississippi in 1870 and tells the story of a swindler made to walk the plank after the passengers discover his schemes. Lead singer Boy George laments that he's ‘a man without conviction’ who ‘doesn’t know/how to sell a contradiction.’ He was singing about his tumultuous relationship with closeted drummer Jon Moss.

While ‘Jeffrey C. Alexander’ would continue to battle for neofunctionalism well into the mid-1990 s, Jeff was faced with a complex of crises. Parsons could not be purified by bringing him back to the mainstream – the critics would make sure of that. At the same time, not enough of Parsons’ concepts could be lashed together into more than just a life raft for Jeff's own sociology – he would sink alongside him, never truly becoming his own theorist. And then the guilt: all the nurturant relationships that helped make neofunctionalism come to life could be lost. How could Jeff face the people who had championed and supported him? Jeff was not swindling his field nor his friends, but the sense of karmic retribution still hung over his head. All of this was ‘[an] excruciating crisis… extremely difficult’ (Alexander, 2020: 75), a slow-motion disintegration of his persona while neofunctionalism crept towards the edge of the plank.

At the very least, Jeff's intellectual break had a starting point in Durkheim and the environments of action. But the reintegration of a creative self would take more than that – better to think with others than by yourself. So in academic year 1984–1985, Jeff offered a new class for graduate students at UCLA:

**245. Cultural Studies.** Discussion, three hours. Prerequisite: consent of instructor. The course allows graduate students to become familiar with the range of contemporary studies of culture. Taking as its assumption that symbolic patterns have relative autonomy vis-à-vis social structure and personality, it examines hermeneutical, structuralist, and functionalist versions of this general argument. It proceeds subsequently to contemporary versions of “interpretive” techniques: structuralist, functionalist, Weberian, Durkheimian, and semiotic. Theory, method, and substantive empirical issues are simultaneously addressed. Mr Alexander (UCLA General Catalog, 1984−1985, 1984: 287)

We can see a step away from Parsonian ‘values’ here − *symbolic patterns* have relative autonomy. With the variety of perspectives in the course, Jeff wished to take his students thoroughly through ‘the cultural turn’ (Hunt and Bonnell, 1999). Although preceded by a linguistic turn in philosophy decades prior with Austin and the later Wittgenstein, the cultural turn was an effect of the romantic counter-culture of the 1960s − which, in the process of reforming a host of institutional spheres across the West (Watts 2022) eventually seeped into the social sciences. By the 1960s and 1970 s, the cultural turn led to a great revival of what Gouldner (1973) once referred to as the Romantic tradition (as opposed to Classicist tradition) in sociology − with its humanistic concerns for subjectivity, consciousness, interpretation, historical contingency and meaning.

In many ways, this was a cultural *re*turn for Jeff. The Marxism of his past was humanistic and cultural, and even before that Mr. Holtby had taught Jeff the close reading techniques of the New Criticism and instilled in him a love of literature. The next several years would see Jeff incorporate new ideas from non-Parsonian cultural arguments: hermeneutics (Dilthey, Gadamer Ricoeur); phenomenology (Heidegger, Schutz Merleau-Ponty); cultural history (Sewell and Hunt); literary theory (Frye); social and cultural anthropology (Douglas, Turner, Sahlins, and Geertz); semiotics and structuralism (Saussure, Levi-Strauss, and Barthes); and post-structuralism and cultural studies (Foucault, Derrida, Lyotard, Hall, Williams, and Thompson). With the help of good friend and interlocutor Steven Seidman, Jeff curated a collection of these ideas in the volume *Culture and Society: Contemporary Debates* (Alexander and Seidman, 1990). In his introduction Jeff writes, ‘recent developments in cultural studies converge in their emphasis on the autonomy of culture from social structure’ (Alexander and Seidman, 1990: 25).^8^ Only implicit here is the idea of a research programme: ‘culture cannot be studied within the framework of a particular school…’ and ‘the differences… point to the need for a more general perspective…’ (Alexander and Seidman, 1990: 26).

Still, the breadth of Jeff's course attracted students with different interests − some for theory, some from UCLA's strong microinteractionist contingent, others from its comparative and historical wing. For the ones who were keenly interested in culture, Jeff arranged a study group to supplement the course. That group included Elaine Chan, Laura Desfor Edles, Anne Kane, Hannah Kully and Eric Rambo; who were soon joined by Jinlin Hwang, Ron Jacobs, Agnes Ku, Andy Roth, Steve Sherwood and Phil Smith in the subsequent year. The study group − which included Jeff − called themselves the ‘Culture Club.’ Instead of meeting in Haines Hall where Sociology was housed, they met once a month in each other's apartments and living rooms off-campus − the Culture Club was the ‘underground’ cousin to the ‘mainstream’ programme of neofunctionalism. Instead of having to explain himself to a hostile professional audience, now Jeff could try out his own cultural turn with likeminded people, and they with him. The spirit was convivial and intellectually vibrant; the members suggested additional readings from outside the social sciences. By the time Jeff returned from Princeton in 1986, the Culture Club members had evolved the group into a workshop for their papers and then their dissertations. The members’ empirical cases were broad and international in scope: Spain, Taiwan, Hong Kong, Ireland, the Black public sphere, American politicians, private schools, the Falklands War, to name a few. Their *cri de coeur* was ‘That's insufficiently cultural!:’ a paper, an idea did not get at meaning and how meaning mattered in a deep enough way.^9^

To get towards that desired ‘sufficiency,’ the Culture Club invested in each other's creativity, synthesizing Jeff's teaching and thinking with their own insights. Durkheim combined with semiotics showed the importance of ritual and codes in meaning-making (e.g., Alexander, 1988a, 1990, 1992; Alexander and Smith, 1993; Edles, 1993 ; Ku, 1998 ; Smith, 1991 ). Action could be read as text (Ricoeur, 1971), but what is more is that text could be read with its narrative genre in mind, explaining solidarity as romance and disenchantment as tragedy and irony (e.g., Alexander, 1995; Jacobs, 1996 ; Sherwood, 1994 ). It was Eric Rambo and Elaine Chan who coined the term ‘culture structures’ in an article published in 1990, finally giving a name to the ‘what’ of culture that had eluded even Jeff (Rambo and Chan, 1990 ). Culture structures ‘constitute’ meaning, Rambo and Chan (1990: 648) argued, and by treating them first as ‘analytically autonomous’ one could reconstruct those meanings via thick description (Kane, 1991).

With core concepts, a methodology and a community the Culture Club had become a crèche for a new kind of cultural sociology. But while his students were confident in their new sociology, Jeff still needed to be unburdened from his old one. Visiting Bernard Giesen, who had become a close friend and intellectual kinsman, Jeff confessed that he had turned away from ‘mainstream sociological theorizing and entered into the opaque, mysteriously exciting world of cultural theory and social meaning’ (Alexander, 2021). To Jeff's relief and surprise, Bernard had also made the same move towards culture that he had: ‘We talked almost in whispers about this secretly shared cultural turn, and our ambition to make use of it to radically change sociology’ (Alexander, 2021).

In 1993–1994, Jeff and his family spent a sabbatical year in France, living in Paris and teaching at the University of Bourdeaux, then at the École des Hautes Études des Sciences Sociales et les Instituts d’Études Politiques. While in Paris, Jeff was integrated with Alain Touraine's group. Perhaps as Touraine suggested, groups could be actors as much as individuals; but like Giddens and other theorists, Touraine had conflated actors with a normative kind of agency that lionized their resistance to social structure (Alexander, 1993). Jeff also began an intense review of the work of Pierre Bourdieu who was on the ascent in the United States, but surprisingly ‘omnipresent but passé’ in discussions in France (Alexander, 1995: 128fn1). In both of these projects the relative autonomy of culture took pride-of-place. It was then that Jeff decided that he could not simultaneously work on neofunctionalism and cultural sociology (Cordero et al., 2008: 526). But the move needed to be explicit: it was on a long walk in Paris that Ron Eyerman and his ‘enlightened friendship’ moved Jeff to go public with the break (Alexander, 1998a: xv).

With his students and close friends behind him, a new ‘Jeffrey C. Alexander’ took the stage. ‘The Reality of Reduction: The Failed Synthesis of Pierre Bourdieu’ on its face looks like the polemical criticisms Jeff delivered in years past. Indeed, the hallmarks of the ‘Jeffrey C. Alexander’ review are here−Bourdieu is ‘irredeemably flawed’ (Alexander, 1995: 130) and cannot avoid an impoverished rational and collective reading of social life. However, ‘The Reality of Reduction’ is not a one-note review: it is closer in spirit to the 105-page paper Jeff wrote for Bellah that inspired *Theoretical Logic*, and the Durkheim two-parter that lamented the anxiety of being misunderstood. If theoretical presuppositions could bring form to critical content in his past reviews, now Jeff was riffing like a virtuoso: he delivers a high-energy barrage of cathartic criticism, rock-a-billy, fingers striking chord after critical chord for *over eighty pages*. Not only does Bourdieu fail to link micro and macro through habitus, but he also ironically condescends to his subjects through his theory of taste, and he cannot imagine a democratic politics beyond the competitive logic of capitals and fields. Most importantly, Bourdieu as the reigning king of the sociology of culture cannot adequately provide the resources for a cultural sociology: meaning for him is reducible to the dynamics of the fields of power and to the strategic interests of its players.

In other words, a cultural sociology with meaning and democracy at its core can only come if we abandon Bourdieu, Jeff suggests. This symbolic boundary − used to separate those cultural programmes that are sufficiently meaning-centred (‘cultural sociology’) and those that reduce meaning to social structure (‘sociology of culture’) − became the critical standard and a new *cri du coeur* for anyone wanting in on the Culture Club, but also a moral one that promised clarity about the state of the world. For instance, in a review of a reader in British cultural studies, Alexander and his students (Sherwood, Smith, and Alexander, 1993) take the editors to task for their dogmatic neo-Marxist sociology of culture, grasping for straws as the fortunes of Marxist analysis have declined. In *Fin de Siècle Social Theory* (Alexander, 1995), Alexander plays the part of the critical theorist, propounding on the interpretive failures of his positivist and postmodern colleagues. Indeed, the seeds of a political turn in Jeff's work begin to take root here too, engaging normative philosophy (Rawls and Habermas) and democratic theory (Cohen and Arato, and Putnam) (Alexander, 1993, 1997), emphasizing their respective sociological blind spots, along with their failures to appreciate the moral and empirical complexity of modern differentiated societies. There was more at stake than just the value of grand theory, more than the charisma of theory-as-sport, as much as ‘Reality of Reduction’ was a stadium-rockin’ banger. Theory could make culture a partner in democratic reflexivity, not its opponent − this would be the task of Jeff's next phase.

For Parsons and neofunctionalism, a gentler goodbye was needed. The break would need to be final, but it would need emotional care: a swan song for a relationship come to an end with the lessons learned from more than 20 years of intense engagement. *Neofunctionalism and After* compiles the core arguments that made up neofunctionalism and The New Theoretical Movement. Declaring the project ‘completed’ (Alexander, 1998: 5), Jeff reviews its chief successes, especially in securing Parsons’ status as a classical theorist (Alexander, 1998: 12): ‘I mean “classical” in the constructivist sense… according to which the name of an intellectual figure comes to function communicatively as a kind of condensed, shorthand symbol for a complex set of ideas’ (Alexander, 1998: 4fn6). He tells the story about how he came to Parsons in the late 1970s with the help of his mentors as a means of communing with the rest of the classical canon: ‘I found Parsons’ theory something I could think with, but never with it alone’ (Alexander, 1998: 9). But he developed ‘a growing sense that there were fundamental and intrinsic difficulties in Parsons’ theorizing,’ leaving him feeling ‘increasingly impatient’ (Alexander, 1998: 11). Recognizing this, however, was not a sign of failure, but of change. In writing *festschrifts* for Bernard Barber and S.N. Eisenstadt (Alexander, 1991, 1992) both of whom supported the neofunctionalism project, Jeff realized his thinking was changing in the same way theirs did: distance, not continuity marked their mature development as thinkers (Alexander, 1998: 12fn18). And so, ‘his story’ − his story with Parsons − was ‘coming to an end,’ Jeff writes. ‘The narrative shifts to the future and away from the past, to the movement beyond neofunctionalism, from within the movement and outside of it as well’ (Alexander, 1998: 8).^10^

The final chapter, ‘After Neofunctionalism,’ is the more melodic twin to the rhythmic and in-your-face ‘Reality of Reduction.’ If the crowd was left in awe of the sublime destruction of Bourdieu, Jeff performs a beautiful act of creation here.^11^ From beyond the limits of Parsons, Jeff shows us exactly how his thinking has matured, why Parsons must be recognized but how he has evolved past him. On action, Jeff had already made tentative steps away from Parsons in ‘Action and Its Environments,’ but here he brings to bear his criticism of Touraine, Giddens and others on agency. ‘Actors are not simply agents… nor are structures necessarily contradictory’ he writes. ‘If we define action as the movement of a person through time and space, we can see that… every action contains a dimension of free will, or agency’ (Alexander, 1998: 215):

‘There are many ways to express this distinction. In my own work, I suggest that agency is the moment of freedom which occurs between three structured environments, and that two of these — culture and personality — exist ontologically only within the actor… According to this model, actors certainly have knowledge, but it is an error to say — as Bourdieu and Giddens do, following Garfinkel — that actors are “knowledgeable agents” as such. This is an error because the knowledge that actors have does not come from their agency as such but from the cultural environment which surrounds it and transforms it into identity’ (Alexander, 1998: 215).

‘It is society's knowledge’ not the actor's practices *per se* that helps them invent, typify and strategize (Alexander, 1998: 216). In other words, within each of us our wants and needs find their expression in the symbols, codes and narratives we share as collective knowledge. Actors can be petty, clumsy and deluded, to be sure (Alexander, 1998: 213, 217). But personality and culture are not outside of us, but inside of us − *they have been there all along*, Jeff seems to say − and between them and the hard reality of the social structures we create, there is always, *always* a dimension of free will: to feel, to express, to choose among the best available options. If we take culture to be something inside of us that we all share, ‘then, we will understand that action must be seen as a constant process of exercising agency *through, not against*, culture’ (Alexander, 1998: 218, emphasis added). Parsons cannot explain this internal environment with ‘values’ defined from outside of it: only a ‘strong program for cultural sociology’ that ‘would never confuse the analysis of social functions with patterned understandings of actors themselves’ can (Alexander, 1998: 220). And the tension between these analytically-defined ‘values’ cannot explain constant tensions in even seemingly solidaristic societies: ‘The sacralized symbolic categories that construct universalism… can be defined only by publicly contrasting them with the profane motives, relations, and institutions that characterize other kinds of individuals, institutions, and groups’ (Alexander, 1998: 226).

Surprisingly, Jeff ends the chapter with a hedge: ‘These brief discussions can perhaps be seen as a prolegomena for a new form of synthetic social theory’ (Alexander, 1998: 228). But unlike the hedge he made in *Culture and Society* that expressed doubt about where all this would lead, here with the Culture Club, his friends, and a “strong program” emerging, this hedge was less an expression of doubt than it was a promise of his next act to come − from Jeff as ‘Jeffrey C. Alexander,’ and ‘Jeffrey C. Alexander’ as Jeff. Or, in other words, from a new man, a new song: no longer a need to sell a contradiction.

## ‘A kind of social psychoanalysis:’ Reconciling the light and the dark, 2001–2013

Relieved of the weight of neofunctionalism and nourished by his friends and students, Jeff's self recovered. Now at midlife, new ambitions and projects culminated marked a new era of productivity. The first of these ambitions was the establishment of a cultural sociology that met the ambitions of his break with Parsons. This process of demarcation and consolidation reaches a new level in the publication of *The Meanings of Social Life* (Alexander, 2003). The Introduction to *Meanings* reads like C. Wright Mills’ chapter ‘The Promise’ in *The Sociological Imagination* (Mills, 1959 [2000]). Mills begins that famous chapter in an existential way, ‘Nowadays men often feel that their private lives are a series of traps,’ and he ultimately directs us to the ‘seemingly impersonal’ qualities of ‘historical change and institutional contradiction’ (Mills, 1959: 3). The sociological imagination would ‘enable [its possessor] to take into account how individuals… often become falsely conscious of their social positions’ (Mills, 1959 [2000]: 8). By ‘[grasping] history and biography and the relations between the two,’ lessons both ‘terrible’ and ‘magnificent’ proceed when a subject becomes aware ‘of all individuals in [similar] circumstances’ (Mills, 1959 [2000]: 5, 6). Mills’ sociology is grave and powerful, promising insight to distinguish between private troubles and public issues and transcending the discipline's tendencies towards statistical analysis with no theory and theory with no practical insights.

A fan of Mills as a student at Harvard, but now with years of reflection and released from the strictures of Parsonian vocabulary, Jeff's echo of Mills is no less grave but with a decidedly different conclusion. With a lightness and elegance in prose that was almost entirely missing during his neofunctionalist phase, Jeff poses the following to the reader:

‘Modern men and women go about their lives without really knowing why. Why do we work for such a long time every day? Why do we finish one war only to fight another? Why are we so obsessed with technology? Why do we live in an age of scandal? Why do we feel compelled those, like the victims of the Holocaust, who have been murdered for an unjust cause?’

…

‘America and its allies are waging today a war on terrorism. This is said to be necessary and rational, a means to attain the end of safety. Is the war against terrorism only this, or even primarily this? No, for it rests on fantasy as much as on fact. The effort to protect the people of the United States and Europe is shrouded in the rhetoric of good and evil, of friends and enemies, of honor, conscience, loyalty, of God and country, of civilization and primeval chaos. These are not ideas. They are feelings, massive ones. Our leaders evoke these rhetorics in solemn tones, and we honor the victims of terrorism in the most rhetorical of benedictions’ (Alexander, 2003: 3).

He draws on his UCLA students’ insights: ‘These rhetorics are cultural structures,’ Jeff writes. ‘The problem is that we don’t understand them’ (Alexander, 2003: 3). And, reminding us of the environments of action, Jeff writes, ‘The secret to the compulsive power of social structures is that they have an inside. They are not only external to actors but internal to them. They are meaningful’ (Alexander, 2003: 4). Jeff's new promise to those seeking answers to these existential questions is cultural sociology, that it could provide ‘a kind of social psychoanalysis… to bring the social unconscious up for view. To reveal to men and women the myths that think them so they can make new myths in return’ (Alexander, 2003: 4). Where Mills’ terrible and magnificent lessons pit the possessor of the sociological imagination against impersonal structures and the weight of history, Jeff's teaches us the possibilities of self-actualization and agency through the internal meanings that animate all of social life.

*Meanings* also served as a definitive programmatic statement for Jeff's new cultural sociology. In the opening chapter to *Meanings*, Jeff provides a genealogy of the Strong Program in Cultural Sociology which roots it in Weber's hermeneutical investigations in the sociology of religion, and in Durkheim's last greatest work, *The Elementary Forms of Religious Life*.^12^ Then, in the second, co-written, chapter, Alexander and Smith elaborate on the distinction between cultural sociology and the sociology of culture by mounting sweeping broadsides against all adjacent cultural programes − for example, British cultural studies, Bourdieusian practice theory, Foucault and post-structuralism and the production and reception of culture approaches − which they view as failing to respect culture's autonomy, and thus betraying the promise contained in the work of the later Durkheim.^13^ At the same time, Alexander and Smith articulate the theoretical bases for their structural hermeneutics − a form of cultural sociology which emphasizes the binary and structural character of symbolic forms, while also insisting on the need for thick hermeneutic reconstructions thereof. It was the publication of *Meanings* that made the Strong Program in Cultural Sociology a recognizable research programme, capable of inspiring the kind of empirical work which Alexander, in his earlier period, largely eschewed.

Jeff's move from UCLA to Yale University in 2001 helped to institutionalize these shifts in scholarly and creative direction. The creation of the Center for Cultural Sociology (CCS) at Yale in 2003 was pivotal: it would become a ‘pilgrimage site’ (Jacobs, 2019: 260) for any and all scholars interested in meaning-centred sociology. By hosting faculty, postdoctoral and doctoral fellows, and holding a weekly workshop, CCS gave institutional form to the informal discussions of the UCLA Culture Club. The creation in 2012 of the *American Journal of Cultural Sociology* − a journal that Jeff co-edits, along with Philip Smith and Ronald Jacobs – has provided a global platform for the Strong Program to demonstrate its utility and value to the wider sociological community. Meanwhile, Jeff and his colleagues continue to edit a book series with Palgrave, which publishes cutting-edge work inspired by the Strong Program.

Indeed, in his role as an ‘elder statesman of sociology’ (Vandenberghe, 2019: 16), Jeff has embraced a more diplomatic role, engaging in far less hostile polemic. To be sure, this stems from having successfully established a research programme whose tenability and intellectual integrity is no longer in doubt. Between 2003 and 2013, the Strong Program would go on to produce a wide array of empirical case studies and theories of the middle-range. These include: the theory of cultural trauma, a Strong Program approach to social suffering, collective memory and collective identity formation (Alexander, 2012); the theory of social performance, an attempt to update the later Durkheim's theory of ritual with insights from dramaturgy and performance theory (Alexander et al., 2006); and the theory of iconic consciousness and iconicity, a Strong Program attempt to theorize the cultural dimensions of the material world (Alexander et al., 2012). Furthermore, by editing a range of book series in cultural sociology (with Cambridge University Press, then Paradigm Publishing and then Palgrave), publishing the *Oxford Handbook in Cultural Sociology* (Alexander et al., 2012) and continuously refining his ideas and responding to critiques of the Strong Program (e.g. Alexander, 2005), Jeff did much in these years to legitimate his distinctive research programme in the eyes of the sociological community.

The second culminating ambition was the 2006 publication of *The Civil Sphere* – Jeff's nearly 800-page *magnum opus*, full stop. *The Civil Sphere* took a long time to write, always present underneath and besides the neofunctionalism project and crèche twin to cultural sociology. Like his Culture Club students, Jeff was fascinated by the ‘Third Wave of Democracy’ in the 1980s and 1990s (Huntington, 1991). The return of explicit references to ‘civil society’ during this time, inspired Jeff to attempt new permutations and engagements with democratic theory (e.g., Alexander, 2000b, 2000c; Alexander and Smith, 1993). Inspired by Durkheim's work on structural differentiation, moral individualism and civic solidarity, by Parson's notion of the societal community, by Bellah's account of civil religion, by T.H. Marshall's ideas of citizenship and social rights and by Michael Walzer's normative-cum-hermeneutic theory of spheres of justice, Alexander retheorizes civil society by producing a novel sociological account of liberal democracy as an institutionalized reality and regulative ideal.

As much a tremendous work of theory, *The Civil Sphere* is an expression of moral and political clarity, drawing a line from the Civil Rights Movement to Solidarity to Watergate to Solidarity and Tiananmen Square, all linked together in their demands for justice and civil repair. ‘We live in a cynical age,’ Alexander writes. But ‘feelings for others matter, and they are structured by the boundaries of solidarity… Solidarity is possible because people are oriented not only to the here and now but to the ideal, to the transcendent, to what they hope will be everlasting’ (Alexander, 2006: 1). In the ensuing years, *The Civil Sphere* would morph from being the prodigious work of a single author to being the basis for a vast and enterprising global research network (Kivisto and Sciortino, 2022). Jeff continues to play a leading role in further developing the theory (Alexander, 2019; Alexander and Tognato, 2017; Alexander et al., 2019a, Alexander et al. 2019b, Alexander et al., 2019c).

The third culminating ambition is less a single text, but a series of interventions that reflected Jeff's renewed and confident engagement with his Jewishness and the sociological lessons of the Jewish diaspora. The child of secular Jews, Jeff's Jewishness had always been a part of his personal life, it was not part of his intellectual output. Through his UCLA period, Jewishness and Judaism posed important questions for Jeff and his family. Deciding that their sons should have the option of choosing a life with faith, Jeff and his family attended Kehillat Israel, a Reconstructionist synagogue in Pacific Palisades, California. An antisemitic incident at UCLA spurred Jeff to co-author several pieces with UCLA Hillel Rabbi Chaim Seidler-Feller (see e.g., Alexander and Seidler-Feller, 1991). These relationships would inspire the notion of civil repair in *The Civil Sphere*: ‘The Jewish phrase Tikkun Olam [kept] coming up in personal talks with my Rabbis in Los Angeles,’ Jeff recalls. ‘Both Rabbis emphatically insisted the phrase did NOT mean “charity,” but rather something akin to social improvement, help, perhaps even “good trouble.”.’

But in his midlife, Jeff would find a deep intersection of his identity, his work on cultural trauma, the modes of incorporation in the civil sphere and the contemporary rise in antisemitism. In the magisterial article, ‘On the Social Construction of Moral Universals,’ Jeff asks ‘How did a specific and situated historical event, an event marked by ethnic and racial hatred, violence, and war, become transformed into a generalized symbol of human suffering and moral evil…?’ (Alexander, 2002: 5). In the final two substantive chapters of *The Civil Sphere* – ‘Anti-Semitism and the Failure of Assimilation’ and ‘Before and After the Holocaust’ – Jeff tests his own theory of incorporation by tracing the exclusion of the Diaspora towards a public, multicultural identity as exemplified by the characters in Philip Roth novels and Woody Allen films. Recently, Jeff has been concerned with the wave of antisemitism that has come at the expense of Holocaust memory (Alexander and Adams, 2023). These engagements with the history of the Jewish Diaspora, Holocaust trauma and antisemitism were also the stories of his parents’ assimilation, Jeff's own experience with hyphenation and the horizon of hope in multicultural America. If culture surrounds us and gives us our identity, as Jeff once wrote, Jewish culture could finally surround Jeff's intellectual action and transform it into *his* identity.

## Conclusion

We started by framing our case for Jeffrey C. Alexander as a classical theorist against and with the structural and cultural arguments for canonization. The structural case can be made by locating Jeff at the centre of networks of scholars, and for at least two − the Strong Program and the Civil Sphere Network − this is definitely true. More subtly, Jeff's work can be found at the roots of more general sociological arguments − the over 9000 pieces that have been cited Mustafa Emirbayer and Ann Miche's ‘What is Agency? (Emirbayer and Miche, 1998) are by extension building on ‘Action and Its Environments.’ The cultural case might simply be to lay out the analogic associations with ‘Jeffrey C. Alexander:’ theory::cultural sociology:: neofunctionalism − for which neofunctionalism retains a dialectical quality in the United States, but not necessarily for the rest of the world. We can conclude that a classical theorist is a symbolic reference; they are a culture structure at the heart of a field. At the core of a research programme, the totemic works of the classical theorist are challenged and extended − their iconic power flowing from object to audience and back again. So, a canonical text one must read, so to Alexander we therefore must go. A canonical argument one must defend, so to Alexander we therefore must commit − especially where we know he is wrong (see Lakatos, 1968–1969).

But our case for canonization traced Jeff's personality development from his early years as a young man looking to prove himself intellectually, to the intellectual and emotional tensions of his early career, through his midlife transition which coincided with his move from neofunctionalism to cultural sociology, and then his late adulthood as a renowned senior scholar and mentor to many. We interpreted some of Jeff's important works as productive breakpoints in Jeff's personal development − these pieces marking movement not just between intellectual phases, but phases of his life. These moments are energetic and prolific, reformulating the self via intellectual action. Indeed, Jeff's work post-2001 evinces a reconciliation of the professional and personal selves which had always been tensely intertwined since his young adulthood, with cultural sociology, civil sphere theory and his explorations in cultural trauma as reflections of newly felt freedoms in creativity, politics and identity, respectively. Philip Smith had identified that many Durkheimian scholars had their most productive years after the age of 45 (Smith, 2023: 415). From our look into Jeff's life, we could add that it is personality transition that matters for this productivity. And, last but not least the great works that moved Jeff from phase to phase were nurtured by new emotional−intellectual environments, by friendships, by being mentored and by mentoring in turn.

We have tried to demonstrate Jeff's humanity as it can be found in the evidence of his existence in this very real world. What are our classical theorists without their human qualities? Weber's pessimism, Marx's restlessness, and Durkheim's anxiety of being misunderstood. And who were they without Marianne, without Frederich and without Marcel? The saints too were human − at least some of them were − but it is their lives as corporeal beings, affected by and oftentimes challenged by their human existence that matters. Many suffered, some were petty, others received signs and gifts and struggled to accept them. But, as the saints, we turn to them when we are in need of their intercession − precisely because their lives might be so much like ours, as much as their gifts of grace set them apart.

## Notes

1. While most of the material used to craft this biographical narrative is publicly accessible (especially Alexander, 2018), some of it derives from various interviews and personal communication from 2023 and 2024.

2. In a longer version of this interview published in the *Harvard Crimson* (Alexander, 1967b), Morrison explains to Alexander that what he and the Doors do goes back to ‘the history of drama in Greek culture’ and the origins of audience and acting − surely a deep influence on cultural pragmatics nearly four decades later. When Jeff asks about the Vietnam War, Morrison replies, ‘War doesn’t exist… Like I don’t believe there is a war’ to which John Densmore says, ‘Really?’

3. See Alexander, ‘The Sixties and Me’ (Alexander,^2005^), esp. pp. 45–46 for Jeff's recollections of this period. And again, we supplement that document with his personal communications with us.

4. In a *festschrift* for Smelser that Alexander would co-edit, he and his co-editors write, ‘it has been Smelser's penchant for combining opposites—the acceptance of sociological ambivalence without fear or favor—that has perhaps most distinctively marked his intellectual career’ (Alexander et al., 2004: 6).

5. In his eulogy for Bellah, he describes him as the father of cultural sociology (Alexander, 2014).

6. Bellah felt a sense of ‘fatherly pride’ when *Theoretical Logic* was published (Bortolini, 2021: 244).

7. In 1989, Jeff received the University of California, Los Angeles Division of Honors Teaching Prize.

8. The phrase ‘relative autonomy of culture’ first appears in Jeff's work in his review of George Homans’ autobiography (Alexander, 1986a: 446, see also 451). Formulations of the phrase appear six times in the introductory chapter to *Culture and Society: Contemporary Debates* (Alexander and Seidman, 1990: 3, 5, 8 thrice, 22), not including the title, ‘Analytic Debates: Understanding the Relative Autonomy of Culture.’ The reader will notice the sentence quoted above does not include the word ‘relative:’ ‘Approaches to culture differ from one another in describing precisely what such autonomy implies’ (Alexander and Seidman, 1990: 25), or rather, why culture is relatively autonomous is relative to the approach − a hedge rather than a declaration. Still, the reason why ‘Analytic Debates’ and not the Homans review marks the beginning of ‘relative autonomy of culture’ was its proto-programmatic intentions.

9. Thanks to Anne Kane, Ron Jacobs and Phil Smith for describing those early meetings in personal communications with us. Phil Smith notes that ‘insufficiently cultural’ replaced ‘not multidimensional:’ ‘I think it was more flexible as it enabled us to make sense of work like Michael Mann's *Sources of Social Power* Vol. 1 [1984] (he was a player in the University of California, Los Angeles department) which was multidimension insofar as he talked about political, economic, military and ideological power’ (personal communication). Jeff remembers the phrase ‘insufficiently cultural’ as ‘that's not cultural enough!’

10. Jeff writes, ‘In referring to Parsons in quotation marks, as “Parsons,” I mean to refer to this constructed figure rather than to the works of the man himself’ (Alexander, 1998: 4fn6).

11. On the importance of destruction/creation polarity at the mid-life transition, see Levinson (1978: 221–228).

12. Of course, this narrative reconstruction is critical for legitimating the disciplinary status of Alexander's project, and thus typifies the strategy of argumentation which, in an earlier period, he describes as constitutive of social scientific debate (Alexander, 1987b).

13. It merits mentioning that all these programmes were framed by Alexander in the 1990s as kindred spirits which contributed to shoring up sociological interest in culture.

14. Thanks to Paul Colomy for this insight.
